# Association between hyperglycemia and the development of chemotherapy induced peripheral neuropathy among patients with breast cancer in the control trial

**DOI:** 10.1186/s13058-025-01984-0

**Published:** 2025-08-18

**Authors:** Miriam Pearl Klahr, Khadija Faheem, Rohit R. Raghunathan, Margaux Wooster, Dawn L. Hershman, Melissa K. Accordino

**Affiliations:** 1 Department of Medicine, Columbia University Medical Center, New York, NY USA; 2 Division of Hematology/Oncology, Department of Medicine, Columbia University Irving Medical Center, New York, NY USA; 3 Department of Biostatistics, Columbia University Medical Center, New York, NY USA; 4 Columbia University Irving Medical Center, 630 W. 168th St, New York, NY 10032 USA

**Keywords:** Breast cancer, Chemotherapy induced peripheral neuropathy (CIPN), Hyperglycemia

## Abstract

**Background:**

Poorly controlled diabetes is a predictor of chemotherapy induced peripheral neuropathy (CIPN) and hyperglycemia may mediate CIPN risk. We evaluated the association between hyperglycemia and CIPN development.

**Findings:**

Methods: This was a secondary analysis of the CONTRoL trial, which randomized patients with Stage I-III breast cancer receiving taxane chemotherapy to cryotherapy, compression therapy, or placebo. CIPN data was obtained from the FACT-NTX survey which patients completed at baseline, week-12, and week-24. Hyperglycemia (glucose ≥ 140 mmol/L) was assessed using random glucose values obtained throughout trial duration. We included patients who completed FACT-NTX at baseline and week-12. Patients were divided into two groups based on CIPN development. For each group we calculated the proportion of patients with hyperglycemia and mean glucose values; 95% confidence intervals were constructed.

**Results:**

Fifty-nine patients met inclusion criteria; 34 patients developed CIPN and 25 did not. Hyperglycemia occurred in 47.1% vs. 36.0% of patients who developed CIPN vs. those who did not (*p* = 0.56). Mean glucose was numerically but not significantly higher in patients with CIPN compared to those without at all timepoints, most notably at week-12 (132.2 mg/dL vs. 122.9 mg/dL *p* = 0.47).

**Conclusions:**

In this analysis, hyperglycemia affected almost half of patients with CIPN versus about a third of patients without CIPN. Patients with CIPN also had higher mean glucose levels compared to those without CIPN, though the differences were not significant. No conclusions can be drawn from these results and further research is needed to assess these trends in a larger prospective patient population.

## Introduction

Chemotherapy induced peripheral neuropathy (CIPN) is a common side effect of taxane chemotherapeutic agents with up to 81% of patients self-reporting symptoms [1]. Symptoms generally include numbness, pain, and temperature sensitivity localized to the peripheral extremities in a glove and stocking distribution. The morbidity can be especially severe, often persisting for years after chemotherapy completion, limiting daily function and thereby impacting patient quality of life [2]. There are limited interventions shown to be successful for the prevention and treatment of CIPN, with duloxetine being the only recommended agent for treatment of painful CIPN [3]. Notably, diabetes is an independent predictor for CIPN development in patients receiving taxane chemotherapy [4]. Moreover, this risk of CIPN development is higher with poorly controlled diabetes, indicating that hyperglycemia may mediate CIPN risk [5]. There is limited research examining the relationship between CIPN and hyperglycemia [6, 7]. Our objective was to evaluate the association between hyperglycemia and CIPN development in patients with breast cancer who participated in the CONTRoL trial [8].

## Methods

This was an IRB approved retrospective study using the database from the CONTRoL trial. The CONTRoL trial was a phase IIB randomized sequential adaptive designed trial of cryotherapy vs. compression therapy vs. placebo for the prevention of CIPN among patients with stage I-III breast cancer and treated with adjuvant or neoadjuvant taxane therapy for at least 12 weeks. Patients completed the Function Assessment of Cancer Therapy Neurotoxicity (FACT-NTX) questionnaire at baseline, week-12 and week-24 of treatment [9]. Success of the intervention was defined as < 5 point change in FACT-NTX from baseline to week-12 based on the literature [8, 10]. Therefore, we defined CIPN development as a > 5 point change in FACT-NTX at week-12. For this analysis, we included patients who completed the FACT-NTX questionnaire at baseline and week-12. Hyperglycemia was defined as any random glucose value ≥ 140 mmol/L, based on the American Diabetes Standards of Medical Care guidelines which define hyperglycemia based on random glucose values (i.e. glucose values not necessarily fasting or prost-prandial) [11]. Hyperglycemia was assessed via chart review of random glucose laboratory results collected from time of taxane initiation through week-24.

Patients were divided into two groups (yes/no) based on CIPN development as assessed from change in FACT-NTX score. Descriptive statistics (mean, median, max, min, IQR) were calculated for demographic clinical characteristics. For each group, we calculated the proportion of patients with hyperglycemia at any point in time. Mean glucose at baseline, +/-7 days of week-12, and +/-7 days of week-24 was also calculated for each group. For all analysis, a p value of < 0.05 was considered statistically significant; 95% confidence intervals were constructed using a t-distribution.

## Results

Sixty-three patients participated in the CONTRoL study and 59 patients were included in this analysis (*n* = 20 cryotherapy, *n* = 21 compression, *n* = 18 placebo). Mean patient age was 53.6 (standard deviation of 13.8); 13.6% of patients were Asian/other; 25.4% were Black, 16% of patients were White and 45.8% of patients were Hispanic/Latino. A total of 74.6% of patients had a BMI ≥ 25 and 13.6% of patients had been previously diagnosed with diabetes mellitus based on review of the electronic medical record. The majority of patients had hormone receptor (HR) positive (55%) breast cancer with 30.5% of patients having stage 1 breast cancer, 50.8% having stage II breast cancer and 18.6% having stage III breast cancer (Table 1). All 59 patients had ≥ 1 recorded glucose value during the 24-week period (*n* = 410 total values), with a median of 6 values (range 1–12) per patient.

**Table 1:** Demographic and clinical characteristics of study population stratified by patients with and without CIPN development

Characteristic *n*(%)	*Overall* *n* = 59	*Patients without Development of CIPN* *n* = 25	*Patients with Development of CIPN* *n* = 34	*P-Value*
Hyperglycemia	25 (42.3)	9 (36)	16 (47)	0.56
Sex				
Female	59 (100.0)	25 (100.0)	34 (100.0)	NA
Age at Consent Mean (SD)	53.64 (13.79)	52.40 (15.73)	54.56 (12.34)	0.56
Race				0.72
Asian/Other	8 (13.6)	4 (16.0)	4 (11.8)	
Black	15 (25.4)	5 (20.0)	10 (29.4)	
Unknown	20 (33.9)	10 (40.0)	10 (29.4)	
White	16 (27.1)	6 (24.0)	10 (29.4)	
Ethnicity				0.69
Hispanic or Latino	27 (45.8)	13 (52.0)	14 (41.2)	
Not Hispanic or Latino	26 (44.1)	10 (40.0)	16 (47.1)	
Unknown	6 (10.2)	2 (8.0)	4 (11.8)	
BMI Kg/m2				0.49
< 25	15 (25.4)	8 (32.0)	7 (20.6)	
>=25	44 (74.6)	17 (68.0)	27 (79.4)	
Clinical Stage				0.79
I	18 (30.5)	7 (28.0)	11 (32.4)	
II	30 (50.8)	14 (56.0)	16 (47.1)	
III	11 (18.6)	4 (16.0)	7 (20.6)	
Receptor Status				0.49
HR Negative/HER2 Negative	17 (28.8)	9 (36.0)	8 (23.5)	
HR Negative/HER2 Positive	9 (15.3)	2 (8.0)	7 (20.6)	
HR Positive/HER2 Negative	20 (33.9)	8 (32.0)	12 (35.3)	
HR Positive/HER2 Positive	13 (22.0)	6 (24.0)	7 (20.6)	
Comorbidities				
Diabetes	8 (13.6)	2 (8.0)	6 (17.6)	0.49
Vitamin D deficiency	8 (13.6)	3 (12.0)	5 (14.7)	1.00
Autoimmune disorder	4 (6.8)	1 (4.0)	3 (8.8)	0.84
Type of Chemotherapy				
Docetaxel every 3 weeks	34 (57.6)	12 (48.0)	22 (64.7)	0.31
Number of docetaxel treatments (Mean (SD))	5.41 (0.9)	5.75 (0.6)	5.23 (0.9)	0.10
Weekly paclitaxel	25 (42.4)	13 (52.0)	12 (35.3)	0.10
Number of paclitaxel treatments (Mean (SD))	11.72 (1.2)	11.46 (1.7)	12.00 (0.0)	0.28
Carboplatin Received				1.00
No	40 (67.8)	17 (68.0)	23 (67.6)	
Yes	19 (32.2)	8 (32.0)	11 (32.4)	
Corticosteroids Received				1.00
No	6 (10.3)	2 (8.3)	4 (11.8)	
Yes	52 (89.7)	22 (91.7)	30 (88.2)	

Of the 59 patients included in this analysis, 34 developed CIPN and 25 did not. When comparing patients who did and did not develop CIPN, demographic and clinical characteristic differences were observed; 29.4% vs. 20.0% were Black (*p* = 0.72), 41.2% vs. 52.0% were Hispanic/Latino (*p* = 0.69), 17.6% vs. 8.0% had diabetes (*p* = 0.49) and 64.7% vs. 48.0% received docetaxel (*p* = 0.31) (Table 1). There were higher numerical but not statistically significant rates of hyperglycemia, 47.1% vs. 36.0%, in patients who developed CIPN vs. those who did not (*p* = 0.56).

**Fig. 1:**
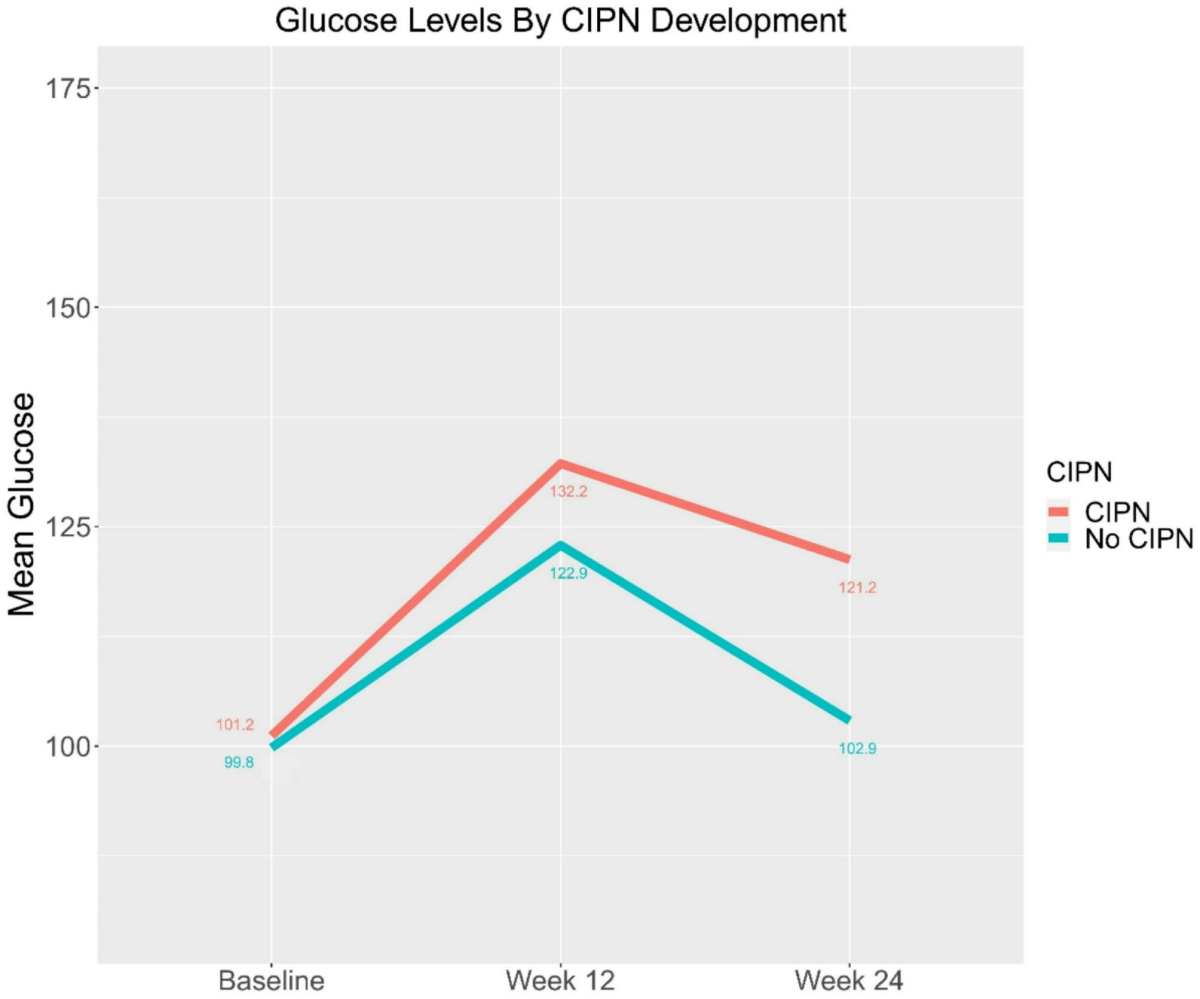
Mean Random Glucose Values at Baseline, Week-12 and Week-24 (*n* = 131 glucose values) Stratified by CIPN Development (*n* = 59 patients)

In addition to evaluating rates of hyperglycemia development, we also evaluated mean glucose values for patients with and without CIPN. Notably, mean glucose was numerically but not statistically significantly higher in patients who developed CIPN compared to those who did not at all timepoints with mean glucose values of 101.2 mg/dL vs. 99.8 mg/dL at baseline (*p* = 0.89), 132.2 mg/dL vs. 122.9 mg/dL at week-12 (*p* = 0.47), and 121.2 mg/dL vs. 102.9 mg/dL at week-24 (*p* = 0.35) (Fig. 1).

## Discussion

This secondary analysis of the CONTRoL trial was a negative study, but notable trends were observed. We found that almost half of patients with CIPN had hyperglycemia compared to about a third of patients without CIPN. Patients with CIPN had higher mean glucose levels at all timepoints compared to patients without CIPN, irrespective of whether their values met the criteria for hyperglycemia. Interestingly, mean glucose values were consistently higher at week-12 compared to week-24, a trend likely attributive to the routine administration of corticosteroids during chemotherapy, with treatment completion occurring before week-24. The differences in the rates of hyperglycemia and mean glucose values between those with and without CIPN were not statistically significant, likely due to the small sample size of our study. This finding is hypothesis generating, but a larger prospective study is needed to determine if hyperglycemia may be an independent risk factor for CIPN development.

Our study has several limitations. All patients received their care at a single urban academic center, which may limit generalizability of our findings. Additionally, there was a small sample size of only 59 patients who met eligibility criteria, limiting the statistical significance of our findings. There was also a differential amount of data between patients and all assessed glucose values were random, including both fasting and non-fasting values, further limiting the generalizability of our findings. Moreover, there is ambiguity about the definition of hyperglycemia based on random glucose levels, as most definitions are based on either fasting or post-prandial glucose values, and due to the nature of this study, all glucose values in this analysis were random. To address this limitation, we used the American Diabetes Standards of Medical Care guidelines which define hyperglycemia based on random glucose values ≥ 140 mmol/L and best fit the data available in this study. We acknowledge that using this threshold for a single random glucose value to define hyperglycemia is not as reliable as when using fasting or two-hour post prandial glucose values, however the latter data was not available for this analysis.

Additionally, our classification of CIPN development was based on FACT-NTX scores, a patient reported outcome (PRO). While there are numerous modalities to assess CIPN in clinical trials, many randomized cooperative group trials use a PRO as the primary endpoint [12–17]. PROs, and specifically FACT-NTX scores, correlate with quantitative sensory tests and are the most meaningful to patients [1]. This was the justification for the primary endpoint for the CONTRoL trial and by extension this secondary analysis. Furthermore, it is important to acknowledge that patients included in the study were actively receiving differing taxane regimens with varying treatment schedules during trial duration, which could also impact FACT-NTX scores. Finally, though the CONTRoL study was a randomized trial, this secondary analysis was retrospective, and our results may be impacted by confounding variables and selection bias.

In summary, we found that there was a higher proportion of hyperglycemia and higher mean glucose among patients who developed CIPN, though this finding lacked statistical significance. Further prospective research with a larger sample size is needed to confirm this finding and demonstrate that hyperglycemia is an independent risk factor for CIPN development.

## Data Availability

The datasets used and/or analyzed during the current study are available from the corresponding author on reasonable request.

## Abbreviations

CIPN: Chemotherapy Induced Peripheral Neuropathy
PRO: Patient reported outcome

## References

[1] Hershman DL, Weimer LH, Wang A, Kranwinkel G, Brafman L, Fuentes D, Awad D, Crew KD. Association between patient reported outcomes and quantitative sensory tests for measuring long-term neurotoxicity in breast cancer survivors treated with adjuvant Paclitaxel chemotherapy. Breast Cancer Res Treat. 2011;125(3):767–74. 21128110 10.1007/s10549-010-1278-0

[2] Armstrong T, Almadrones L, Gilbert MR. Chemotherapy-induced peripheral neuropathy. Oncol Nurs Forum. 2005;32(2):305–11. 15759068 10.1188/05.ONF.305-311

[3] Smith EM, Pang H, Cirrincione C, Fleishman S, Paskett ED, Ahles T, Bressler LR, Fadul CE, Knox C, Le-Lindqwister N, et al. Effect of Duloxetine on pain, function, and quality of life among patients with chemotherapy-induced painful peripheral neuropathy: a randomized clinical trial. JAMA. 2013;309(13):1359–67. 23549581 10.1001/jama.2013.2813 PMC3912515

[4] Gu J, Lu H, Chen C, Gu Z, Hu M, Liu L, Yu J, Wei G, Huo J. Diabetes mellitus as a risk factor for chemotherapy-induced peripheral neuropathy: a meta-analysis. Support Care Cancer. 2021;29(12):7461–9. 34085148 10.1007/s00520-021-06321-7 PMC8550712

[5] Hershman DL, Till C, Wright JD, Awad D, Ramsey SD, Barlow WE, Minasian LM, Unger J. Comorbidities and risk of Chemotherapy-Induced peripheral neuropathy among participants 65 years or older in Southwest oncology group clinical trials. J Clin Oncol. 2016;34(25):3014–22. 27325863 10.1200/JCO.2015.66.2346 PMC5012713

[6] Brunello A, Kapoor R, Extermann M. Hyperglycemia during chemotherapy for hematologic and solid tumors is correlated with increased toxicity. Am J Clin Oncol. 2011;34(3):292–6. 20622641 10.1097/COC.0b013e3181e1d0c0

[7] Schneider BP, Zhao F, Wang M, Stearns V, Martino S, Jones V, Perez EA, Saphner T, Wolff AC, Sledge GW Jr., et al. Neuropathy is not associated with clinical outcomes in patients receiving adjuvant taxane-containing therapy for operable breast cancer. J Clin Oncol. 2012;30(25):3051–7. 22851566 10.1200/JCO.2011.39.8446 PMC3732004

[8] Accordino MK, Lee S, Leu CS, Levin B, Trivedi MS, Crew KD, Kalinsky K, Raghunathan R, Faheem K, Harden E, et al. Randomized adaptive selection trial of cryotherapy, compression therapy, and placebo to prevent taxane-induced peripheral neuropathy in patients with breast cancer. Breast Cancer Res Treat. 2024;204(1):49–59. 38060077 10.1007/s10549-023-07172-y PMC10840989

[9] Calhoun EA, Welshman EE, Chang CH, Lurain JR, Fishman DA, Hunt TL, Cella D. Psychometric evaluation of the functional assessment of Cancer therapy/gynecologic oncology Group-Neurotoxicity (Fact/GOG-Ntx) questionnaire for patients receiving systemic chemotherapy. Int J Gynecol Cancer. 2003;13(6):741–8. 14675309 10.1111/j.1525-1438.2003.13603.x

[10] Hershman DL, Unger JM, Crew KD, Minasian LM, Awad D, Moinpour CM, Hansen L, Lew DL, Greenlee H, Fehrenbacher L, et al. Randomized double-blind placebo-controlled trial of acetyl-L-carnitine for the prevention of taxane-induced neuropathy in women undergoing adjuvant breast cancer therapy. J Clin Oncol. 2013;31(20):2627–33. 23733756 10.1200/JCO.2012.44.8738 PMC3699727

[11] American Association of Clinical Endocrinologists and American Diabetes. Association consensus statement on inpatient glycemic control - PubMed. Diabetes Care. 2009 Jun, 32(6). 10.2337/dc09-9029 PMC2681039 19429873

[12] Hershman DL, Unger JM, Crew KD, Till C, Greenlee H, Minasian LM, Moinpour CM, Lew DL, Fehrenbacher L, Wade JL. Two-Year trends of Taxane-Induced neuropathy in women enrolled in a randomized trial of Acetyl-L-Carnitine (SWOG S0715). J Natl Cancer Inst. 2018;110(6):669–76. 3rd et al. 29361042 10.1093/jnci/djx259 PMC6005110

[13] Budd GT, Barlow WE, Moore HC, Hobday TJ, Stewart JA, Isaacs C, Salim M, Cho JK, Rinn KJ, Albain KS, et al. SWOG S0221: a phase III trial comparing chemotherapy schedules in high-risk early-stage breast cancer. J Clin Oncol. 2015;33(1):58–64. 25422488 10.1200/JCO.2014.56.3296 PMC4268253

[14] Leal AD, Qin R, Atherton PJ, Haluska P, Behrens RJ, Tiber CH, Watanaboonyakhet P, Weiss M, Adams PT, Dockter TJ, et al. North central Cancer treatment group/alliance trial N08CA-the use of glutathione for prevention of paclitaxel/carboplatin-induced peripheral neuropathy: a phase 3 randomized, double-blind, placebo-controlled study. Cancer. 2014;120(12):1890–7. 24619793 10.1002/cncr.28654 PMC4047184

[15] Pachman DR, Qin R, Seisler DK, Smith EM, Beutler AS, Ta LE, Lafky JM, Wagner-Johnston ND, Ruddy KJ, Dakhil S, et al. Clinical course of Oxaliplatin-Induced neuropathy: results from the randomized phase III trial N08CB (Alliance). J Clin Oncol. 2015;33(30):3416–22. 26282635 10.1200/JCO.2014.58.8533 PMC4606060

[16] Trivedi MS, Unger JM, Henry NL, Darke A, Hertz DL, Brannagan T, Smith S, Schneider BP, Irvin WJ, Hathaway AR, et al. Risk prediction model for taxane-induced peripheral neuropathy (TIPN) in patients with early-stage cancer receiving taxane therapy: SWOG S1714. J Clin Oncol. 2024;42(16suppl):12005–12005.

[17] Duloxetine to Prevent Oxaliplatin-Induced Chemotherapy-Induced Peripheral Neuropathy. a Randomized, Double-Blind, Placebo-Controlled Phase II to Phase III Study. In. Edited by National Cancer I; 2019.

